# The enhancing therapeutic effect of neonatal jaundice by *bifidobacterium* through regulating inflammation and gut microbiota in combination with phototherapy—a randomized controlled trial

**DOI:** 10.3389/fmicb.2026.1761245

**Published:** 2026-04-01

**Authors:** Feng Zhang, Jiayi Chen, Yanhan Yuan, Juanjuan Chen, Wei Jiang, Weiwei Xiang, Na Wang, Zhimin Wu, Sainan Fan, Kun Zhang, Yanan Ma, Tianyu Liu, Jiahui Zhang, Qinghua Yu, Jinping Zhang

**Affiliations:** 1 College of Food Science and Technology, Shanghai Ocean University, Shanghai, China; 2 Department of Pediatrics, Shanghai Sixth People's Hospital Affiliated to Shanghai Jiao Tong University, DiproX Medical Research Center, Shanghai, China; 3 Laboratory of Microbiology, Immunology, and Metabolism, Diprobio (Shanghai) Co, Limited, Shanghai, China

**Keywords:** bilirubin metabolism, gut microbiota, neonatal jaundice, probiotics, repair of inflammatory injury

## Abstract

**Background:**

Hyperbilirubinemia is among the most common conditions in neonates, and phototherapy is currently the most widely used treatment. However, it can induce side effects such as skin rashes, diarrhea, and gut microbiota dysbiosis, particularly affecting *Bifidobacterium* levels. This study aimed to investigate whether the supplementation of *Bifidobacterium* can alleviate dysbiosis and improve clinical outcomes in jaundiced neonates.

**Methods:**

A total of 79 jaundiced neonates were enrolled and divided into four groups: Phototherapy Control, M-16V, Bb-12, and the combined M-16V+Bb-12 group. Probiotics were administered until 30 days post-discharge, and neurodevelopment was assessed at 1.5–2 years using the Griffith Development Scales. Fecal samples collected before, during, and after treatment were analyzed using metagenomic sequencing and non-targeted metabolomics.

**Results:**

Probiotic supplementation significantly increased daily defecation frequency, accelerated the reduction rate of transcutaneous bilirubin, and shortened hospital stays. Griffith scores indicated that Bb-12 supplementation improved scores in personal-social and performance domains. Metagenomic analysis revealed significant differences in beta diversity between the control and probiotic groups; specifically, M-16V and combined supplementation increased the abundance of *Bifidobacterium* breve. Pathway enrichment analysis showed up-regulation of pyrimidine-containing compound metabolic processes, intramolecular transferase activity, and DNA conformation change. Metabolomics further demonstrated that combined supplementation elevated levels of 5-methyltetrahydrofolate (linked to DNA synthesis), benzoic acid and indoleacetic acid (linked to growth and development), and the anti-inflammatory metabolite indole-3-lactic acid.

**Discussion:**

For neonates receiving phototherapy, the addition of M-16 V + Bb-12 probiotics can improve the diversity of microflora, reduce the fixed value of harmful bacteria in the intestine, and enhance the excretion of bilirubin from the intestine, to improve the inflammatory damage and microbiota disorder caused by phototherapy, and achieve the effect of clinically improving jaundice, reducing bilirubin, shortening the length of hospitalization, and promoting neurodevelopment. It provides a safer and more effective treatment for neonatal jaundice.

## 1 Introduction

Neonatal hyperbilirubinemia is one of the most common diseases in newborns, classified into pathological types (Kemper et al., 2022; Erdeve et al., 2018). With an incidence rate as high as 80–90%, it is the most common cause of readmission in the neonatal period (Olusanya et al., 2018a). If not treated in time, the condition may progress to acute bilirubin encephalopathy and kernicterus, affecting the long-term prognosis of the child. This progression is accompanied by a significant risk of neonatal death and long-term neurodevelopmental disorders (Olusanya et al., 2018b; Liu H.W. et al., 2023). Currently, phototherapy is the most commonly used effective and safe method to reduce serum bilirubin levels and prevent the occurrence of severe hyperbilirubinemia and bilirubin encephalopathy (Yang and Wang, 2023; Neonatology Group of Pediatrics Branch of Chinese Medical Association, Editor of Chinese Journal of Pediatrics commission, 2014). However, phototherapy has certain side effects, such as rash, fever, and water and electrolyte balance (Faulhaber et al., 2019), and these side effects during phototherapy may be related to flora disorders (Feng and Tong, 2020; Tao and Jiang, 2021).

Winston et al. (2021) demonstrated that gut microbiota plays a highly efficient role in metabolizing bilirubin. A stable abundance of intestinal flora is beneficial for the treatment of neonatal jaundice (Zhang et al., 2021). The critical role of the gut microbiota in the development and persistence of intestinal inflammation underscores the importance of microbiota–host interactions in health and disease. Experimental animal studies and clinical data have confirmed the influence of the gut microbiome in ameliorating inflammation, highlighting its potential as a therapeutic strategy for treating inflammatory diseases (Lo et al., 2020). The occurrence of jaundice, as well as the corresponding drugs and treatment plans, may disrupt the intestinal homeostasis, leading to microbiota disorder, and thus affecting health (Fan et al., 2022). Experimental evidence also supports the fact that dysbiosis has been implicated in the etiopathogenesis of inflammatory bowel disease (IBD) (Roy and Dhaneshwar, 2023). Probiotics have gained attention for mitigating dysbiosis in IBD patients (Jadhav et al., 2023). Adding probiotics has been widely used to improve the balance of intestinal flora and regulate intestinal function (Mutlu et al., 2020; Chen et al., 2017). Our preliminary clinical studies have found that the side effects of phototherapy may be related to a flora disorder (Zhang et al., 2022). Among them, the abundance of two probiotics that can be used for newborns decreased significantly after phototherapy: *Bifidobacterium breve* and *Bifidobacterium animalis* (Yang et al., 2019).

However, in these studies, only a single probiotic was usually used. We aimed to investigate whether a combination of probiotics is superior to a single application, whether it reduces the microbiota disturbance caused by phototherapy, whether it reduces the inflammatory response, and whether it is beneficial for the clinical resolution of jaundice in these neonates. Therefore, we hypothesize that the supplementation of these two probiotics will significantly reduce microbiota disturbance and reduce intestinal inflammatory responses during phototherapy. In this study, we set up a combined probiotic with a 30-day follow-up period involving extensive tool samples. Additionally, follow-up using the Griffith Mental Development Scales was performed at 1.5–2 years of age to supplement existing data.

## 2 Methods

### 2.1 Study design

This was a single-center, single-blind randomized controlled trial. The study was conducted by recruiting neonates hospitalized in the NICU of the Lingang Campus of the Sixth People’s Hospital Affiliated to Shanghai Jiao Tong University from 1 September 2020 to 1 October 2023.

Randomization was achieved using a random number table. The random system parameters were set as follows: (1) Random system: Taimei Medical eBalance random system; (2) Random method: block randomization; (3) Block length: 5; (4) Number of blocks: 20; (5) Random number: P001-P100. The operation steps were as follows: The random-related parameters were determined according to the project’s scheme. The eBalance stochastic system is configured according to these parameters. The eBalance random system generated a random grouping table based on the configuration [calling Statistical Analysis System (SAS) software] and exported the random grouping table.

The study was designed to be single-blind. None of the neonatologists were aware of the group assignments throughout the study, while the parents were aware of whether their infant was assigned to the experimental or control group.

The study was registered at http://www.chictr.org.cn/index.aspx (registration number: ChiCTR2000036013) before the first participant was enrolled.

This study was reviewed and approved by the Ethics Committee of the East Hospital of Shanghai Sixth People’s Hospital in compliance with the Declaration of Helsinki and other relevant regulations (Ethics Approval Number 2020-071). All enrolled neonatal parents provided informed consent.

### 2.2 Participants

The inclusion criteria were: (1) Jaundice index: According to the 2022 American Academy of Pediatrics phototherapy guidelines, the jaundice index reached the phototherapy threshold; (2) age ≤2 weeks; (3) Term infants with gestational age ≥37 weeks and <42 weeks, and birth weight ≥2,500 g and <4,000 g; (4) No prior use of antibiotics or ecological agents before specimen collection; (5) Healthy mothers during pregnancy, with no history of special drug use, and no intake of antibiotics or microecological agents before, during, or after childbirth; (6) Enrolled infants were exclusively breastfed, exclusively formula-fed, or mixed-fed before admission; (7) All enrolled infants had neonatal pathological jaundice as defined by “Practical Neonatology” and required hospital admission solely for phototherapy; (8) Informed consent provided voluntarily.

The Exclusion criteria included: (1) Gestational age <37 weeks or ≥42 weeks; (2) bilirubin levels reaching the exchange blood transfusion standard or elevated direct bilirubin; (3) complications with pneumonia, septicemia, or other diseases; (4) patients with severe immunodeficiency diseases; (5) those with inherited metabolic diseases; (6) congenital biliary malformations or other organ malformations; (7) drug allergies; (8) situations that may warrant exclusion as determined by the researcher, such as a guardian with mental illness or frequent changes in living or working environments, which may result in loss of follow-up.

Stool collection was still required after each phototherapy session.

According to the criteria above, a total of 95 neonates with jaundice were screened, 85 met the inclusion criteria, 6 were excluded due to antibiotic use or refusal to follow up, and 79 were finally included in the study.

### 2.3 Intervention

**Figure 1:**
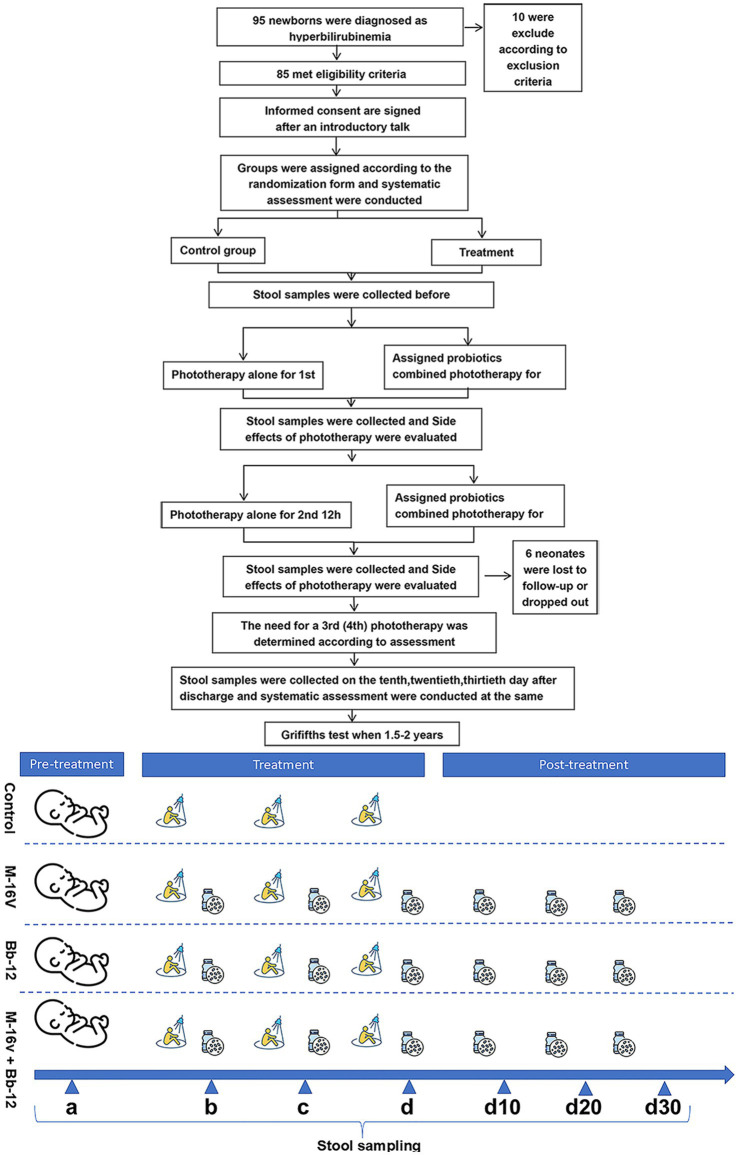
Study design process.

Figure 1 illustrates the details of the research procedure. Once the newborn was confirmed to meet the phototherapy standards for their age, they were registered. Parents continued to receive study information for 30 days, including during their regular follow-up visits to the hyperbilirubinemia clinic.

All the probiotics used in this study were from Dipro, including *B. breve* M-16 V (Dipro M-16 V) and *B. animalis* subsp. lactis Bb-12 (Dipro Bb-12). The probiotic dose is 10^9^ colony-forming units administered orally once daily for 1 month.

The enrolled neonates were divided into 4 groups according to a digital randomization: the control group (pure phototherapy group), the M-16 V group (*B. breve* M-16 V combined with phototherapy), the Bb-12 group (*B. animalis subsp. lactis* BB-12 combined with phototherapy), and the M-16 V + Bb-12 group (M-16 V combined with Bb-12 combined with phototherapy).

The arrangements for enrollment during hospitalization and outpatient follow-up after discharge are shown in Table 1. At the beginning of the study, the investigator collected stool and blood samples from the subjects. Subjects received the assigned probiotics before the first 12 h of phototherapy. After 12 h of phototherapy, researchers recorded information, collected stool samples, and measured transcutaneous bilirubin (TCB). The next day, after 6–8 h of rest, the same process was repeated on the second and third day, and if necessary, subjects continued with the fourth and fifth 12-h phototherapy sessions. Subjects were not required undergo further phototherapy and were discharged once they met the discharge criteria. Follow-up studies on days 10, 20, and 30 were completed, during which stool and related data were collected. Infants in the experimental group continued to receive their assigned oral probiotics for 1 month. When these children were 1.5–2 years old, the Griffiths test was performed, and the results were recorded.

**Table 1:** Inspection schedule.

Time window	Enrollment D0	Phototherapy for 12 h D1	Rest for 6–8 h after phototherapy D1	Phototherapy for 12 h D2	Rest for 6–8 h after phototherapy D2	Phototherapy for 12 h D3	Rest for 6–8 h after phototherapy D3	On the day of discharged D4	Post-discharge D10, D20, D30	1.5–2 years-old
Informed consent	X									
Inclusion and exclusion criteria	X	X	X	X	X	X	X	X	X	
Feeding situation	X	X	X	X	X	X	X	X	X	
Give light therapy intervention		X		X		X				
Random given to probiotics		X		X		X		X	X	
check-up	X	X	X	X	X	X	X	X	X	
Vital sign	X	X	X	X	X	X	X	X	X	
Weight/urine output	X	X	X	X	X	X	X		X	
Serum examination 1		X								
Systemic assessment	X	X	X	X	X	X	X	X	X	
Percutaneous bilirubin determination	X	X	X	X	X	X	X	X	X	
Stool assessment and systemic assessment		X		X		X		X	X	
Grififiths test										X

### 2.4 Sample collection

Stool samples weighing 500 mg and blood samples measuring 0.5 mL were collected from each subject. The stool samples were stored at −80 °C after freezing.

### 2.5 Sample size

A margin of 0.05 was assumed, with a type I error of 0.05 and a power of 0.8. Using a difference test for two-sample ratios, a sample size of 20 cases per group was calculated, yielding a total of 80 cases in both the experimental and control groups.

### 2.6 Metagenomic profiling

#### 2.6.1 DNA extraction and sequencing

The qualified DNA samples were randomly broken into fragments of about 350 bp in length by ultrasonication, and the entire library preparation was completed through end repair, 3′ end A addition, sequencing adapter addition, purification, fragment selection, polymerase chain reaction (PCR) amplification, and other steps. After library construction was completed, the effective concentration of the library was quantified using the quantitative PCR (qPCR) method (library effective concentration > 3 nM) to ensure its quality for subsequent sequencing. Metagenomic DNA was sequenced using a 2 × 150 bp paired-end protocol on Illumina HiSeq.

#### 2.6.2 Sequencing data quality control

Trimmomatic (version 0.39) was used to remove low-quality sequences. Quality control of sequencing reads was conducted to remove low-quality reads and trim low-quality bases. KneadData was used to remove the contamination sequence from human DNA. Before and after removal, FastQC is used to assess sequence quality.

#### 2.6.3 Taxonomy annotation

Host-filtered microbial reads were classified against bacterial, viral, fungal, archaeal, and human genomes using Kraken2 on a reference database constructed from the National Center for Biotechnology Information (NCBI) nucleotide and Reference Sequence (RefSeq) databases. The classification report was then used by Bracken to estimate species abundance, yielding the number of reads per species in the sample.

#### 2.6.4 Functional annotation

Functional analysis was performed using HUMAnN2, based on the UniRef90 database, and annotated with the Kyoto Encyclopedia of Genes and Genomes (KEGG) database to generate KEGG Orthology (KO) and pathway-level profiles for each sample.

### 2.7 Metabolites profiling

#### 2.7.1 Sample preparation

Feces (100 mg) were individually ground in liquid nitrogen, and the homogenate was resuspended in prechilled 80% methanol by vortexing. The samples were incubated on ice for 5 min and then centrifuged at 15,000 *g*, 4 °C for 20 min. Some of the supernatant was diluted to a final concentration of 53% methanol with liquid chromatography–mass spectrometry (LC–MS) grade water. The samples were subsequently transferred to a fresh Eppendorf tube and then centrifuged at 15,000 *g*, 4 °C for 20 min. Finally, the supernatant was injected into the liquid chromatography–tandem mass spectrometry (LC–MS/MS) system for analysis.

#### 2.7.2 LC–MS analyses

Ultra-high performance liquid chromatography–tandem mass spectrometry (UHPLC–MS/MS) analyses were performed using a Vanquish UHPLC system (Thermo Fisher, Germany) coupled with an Orbitrap Q Exactive™ HF mass spectrometer or Orbitrap Q Exactive™ HF-X mass spectrometer (Thermo Fisher, Germany). Samples were injected onto a Hypersil Gold column (100 × 2.1 mm, 1.9 μm) using a 17-min linear gradient at a flow rate of 0.2 mL/min. The eluents for the positive polarity mode were eluent A (0.1% formic acid [FA] in water) and eluent B (methanol). The eluents for the negative-polarity mode were eluent A (5 mM ammonium acetate, pH 9.0) and eluent B (methanol). The solvent gradient was set as follows: 2% B, 1.5 min; 2–85% B, 3 min; 85–100% B, 10 min; 100–2% B, 10.1 min; and 2% B, 12 min. The Q Exactive™ HF mass spectrometer was operated in positive/negative polarity mode with a spray voltage of 3.5 kV, capillary temperature of 320 °C, sheath gas flow rate of 35 psi, and aux gas flow rate of 10 L/min, S-lens Radio Frequency (RF) level of 60, and Aux gas heater temperature of 350 °C.

#### 2.7.3 Data processing and metabolite identification

The raw data files generated by UHPLC–MS/MS were processed using the Compound Discoverer 3.3 (CD3.3, Thermo Fisher, Germany) to perform peak alignment, peak picking, and quantitation for each metabolite. The main parameters were set as follows: peak area was corrected with the first quality control (QC); actual mass tolerance, 5 ppm; signal intensity tolerance, 30%; and minimum intensity, etc. Then, peaks were matched against mzCloud,^1^ mzVault, and MassList database to obtain the accurate qualitative and relative quantitative results, and metabolites were annotated using the KEGG database.^2^ Peak intensities were normalized to the median value per sample, and then log-transformed and normalized with the mean and standard deviation per metabolite.

Normalized data was used for further analysis, such as Principal Component Analysis (PCA, differential analysis), and correlation analysis with species abundance. Statistical analyses were performed using the statistical software R (R version R-4.3.1).

### 2.8 *In vitro* experiment

#### 2.8.1 Anti-inflammatory test

Human adult low Calcium high Temperature cells (HaCaT) (1 × 10^5^/well) were plated on 96-well plates and cultured until adhered. The culture medium was discarded, 100 μL of Dulbecco’s Modified Eagle Medium (DMEM) solution was added to the negative wells (control), and 100 μL of DMEM solution containing 1 × 10^7^ colony-forming unit (CFU) probiotics was added to the sample wells. The plates were incubated for 18 h. The supernatant from the above culture was collected, and interleukin 10 (IL-10) concentration was measured using a kit.

#### 2.8.2 Cell growth test on blue light

After HaCaT cells were seeded into 96- and 12-well plates and cultured for 24 h, the HaCaT cell medium was changed, and Bb-12, M-16 V, and Bb-12 + M-16 V [multiplicity of infection (MOI) = 1:50] were added for treatment. The cells were then exposed to blue light (wavelength 450–460 nm) for 30 m. The control group (control) was treated in the dark under the same conditions. The cells were then cultured in a 37 °C carbon dioxide incubator for 18 h. After the culture, (1) the cells were washed 3 times with phosphate-buffered saline (PBS), and the cell activity was determined by the Cell Counting Kit-8 (CCK-8) method; (2) the cells were collected, and the intracellular malondialdehyde (MDA) level was detected; (3) the supernatant after culture was collected and the tumor necrosis factor-*α* (TNF-α) level was detected using an ELISA kit.

### 2.9 Bioinformatic analysis and statistics

#### 2.9.1 Microbiota diversity analysis

The alpha diversity between groups was analyzed with Student’s *t*-test. The beta diversity was calculated using Bray–Curtis dissimilarity. Permutational multivariate analysis of variance (PERMANOVA) was performed using the “adonis” function in the R Vegan package to assess the effects of phenotype on taxonomic and metabolomic profiles.

## 3 Results

### 3.1 General information

A total of 79 patients were enrolled in the study, and 382 stool samples were collected at multiple time points. There were 20 patients in the control group, 19 in the M-16 V group, 19 in the Bb-12 group, and 21 in the M-16 V + Bb-12 group. No significant differences were found among groups with respect to confounding factors such as delivery mode, gender, age, mothers’ age, gestational age, birth weight, feeding pattern, and percutaneous bilirubin before treatment (Table 2).

**Table 2:** Confound factors of different groups.

Parameters	Control	M-16 V	Bb-12	M-16 V + Bb-12	*p*-value
Patient number	20	19	19	21	–
Delivery mode (natural: C-section)	12:8	14:5	11:8	14:7	0.83
Gender (males: females)	10:10	13:6	12:7	15:6	0.14
Age (days)	4.55 ± 2.82	5.26 ± 2.56	4.37 ± 2.39	5.95 ± 3.20	0.28
Mother age (yeas)	32.00 ± 4.23	31.90 ± 2.96	29.50 ± 5.59	31.2 0± 4.39	0.38
Gestational age (days)	270 ± 9.10	275 ± 6.59	276 ± 7.93	270 ± 8.14	0.12
Birth weight (g)	3,214 ± 435	3,424 ± 498	3,177 ± 304	3,306 ± 379	0.38
Feeding (mix: breastfeeding: formula)	6:5:8	12:3:4	5:10:4	8:9:4	0.08
Pre-treatment skin bilirubin (mg/dL)	15.00 ± 2.79	14.70 ± 2.19	13.50 ± 2.20	14.60 ± 2.90	0.45

### 3.2 Clinical outcomes

**Figure 2:**
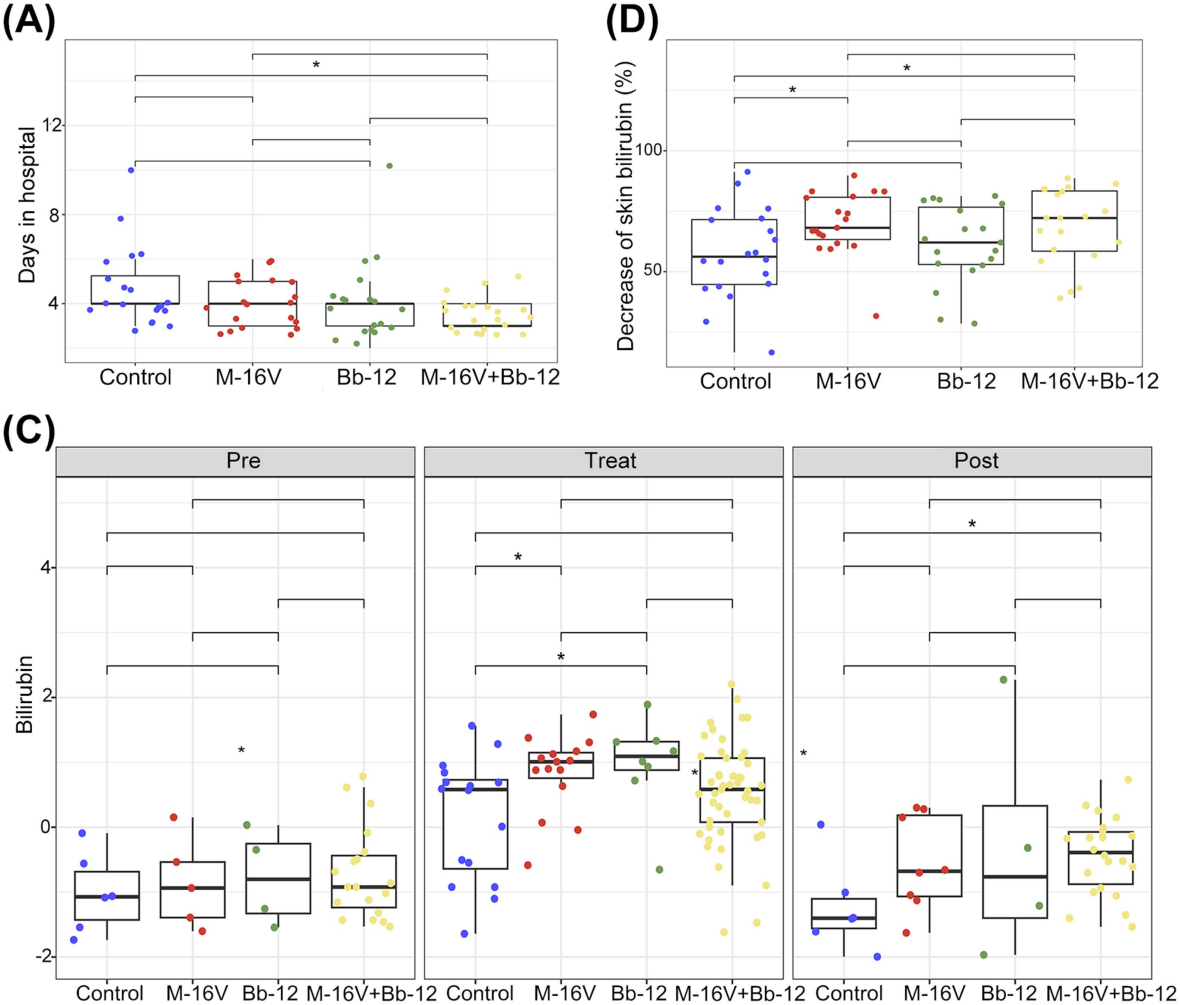
Clinical evaluation of a probiotic supplement on neonatal jaundice with phototherapy. **(A)** Comparison of hospital stay; **(B)** comparison of the reduction rate of skin bilirubin; **(C)** comparison of fecal bilirubin. *p < 0.05.

To compare the efficacy of probiotics for jaundice with phototherapy, we measured the crucial clinical outcomes (Table 3). There was no significant difference in weight change among the groups, whereas probiotics-supplemented groups exhibited higher fecal times per day in treatment and lower skin bilirubin levels after treatment. Specifically, the daily frequency of stool was 3.35 ± 1.18 in the control group, 4.16 ± 1.30 in the M-16 V group, 4.84 ± 1.01 in the Bb-12 group, and 4.33 ± 1.20 in the M-16 V + Bb-12 group (*p* = 0.002, *p* < 0.05). The average length of stay was 4.7 ± 1.81 days for the control group, 4.0 ± 1.05 days for M-16 V, 4.06 ± 1.86 days for Bb-12, and 3.6 ± 0.75 days for the M-16 V + Bb-12 group. Compared with the control group, the length of hospital stay in the M-16 V + Bb-12 group was reduced (*p* < 0.05). Besides, the significant differences in the percentage of skin bilirubin reduction and hospital stay were found between the control and M-16 V + Bb-12 groups (Figures 2A,B), indicating that combined supplementation with M-16 V and Bb-12 may enhance the efficacy of phototherapy and therefore decrease hospitalization time for the newborns. We also measured bilirubin levels in fecal samples at multiple time points. An increased level of bilirubin was observed in the Bb-12 and M-16 V groups during the phototherapy process. At the visiting stage, the bilirubin level in the fecal sample was significantly higher in the M-16 V + Bb-12 combined group (Figure 2C).

**Table 3:** Clinical outcomes of different groups.

Parameters	Control	M-16 V	Bb-12	M-16 V + Bb-12	*p*-value
Patient number	20	19	19	21	–
Weight change (%)	−1 ± 5	1 ± 6.9	2 ± 6	1 ± 5	0.59
Fecal times per day	3.35 ± 1.18	4.16 ± 1.30	4.84 ± 1.01	4.33 ± 1.20	0.002**
Post-treatment skin bilirubin (mg/dL)	6.09 ± 2.09	4.26 ± 1.50	5.21 ± 2.31	4.35 ± 2.08	0.018*
Skin bilirubin loss ratio (%)	57.50 ± 18.70	69.90 ± 13.10	61.30 ± 16.30	68.80 ± 15.70	0.051
Days in hospital	4.7 0± 1.81	4.00 ± 1.05	4.06 ± 1.86	3.60 ± 0.75	0.088

*p < 0.05; **p < 0.01.

### 3.3 The Griffith scale indicated that probiotics have long-term effects on the growth and development of neonates with jaundice after phototherapy

**Figure 3:**
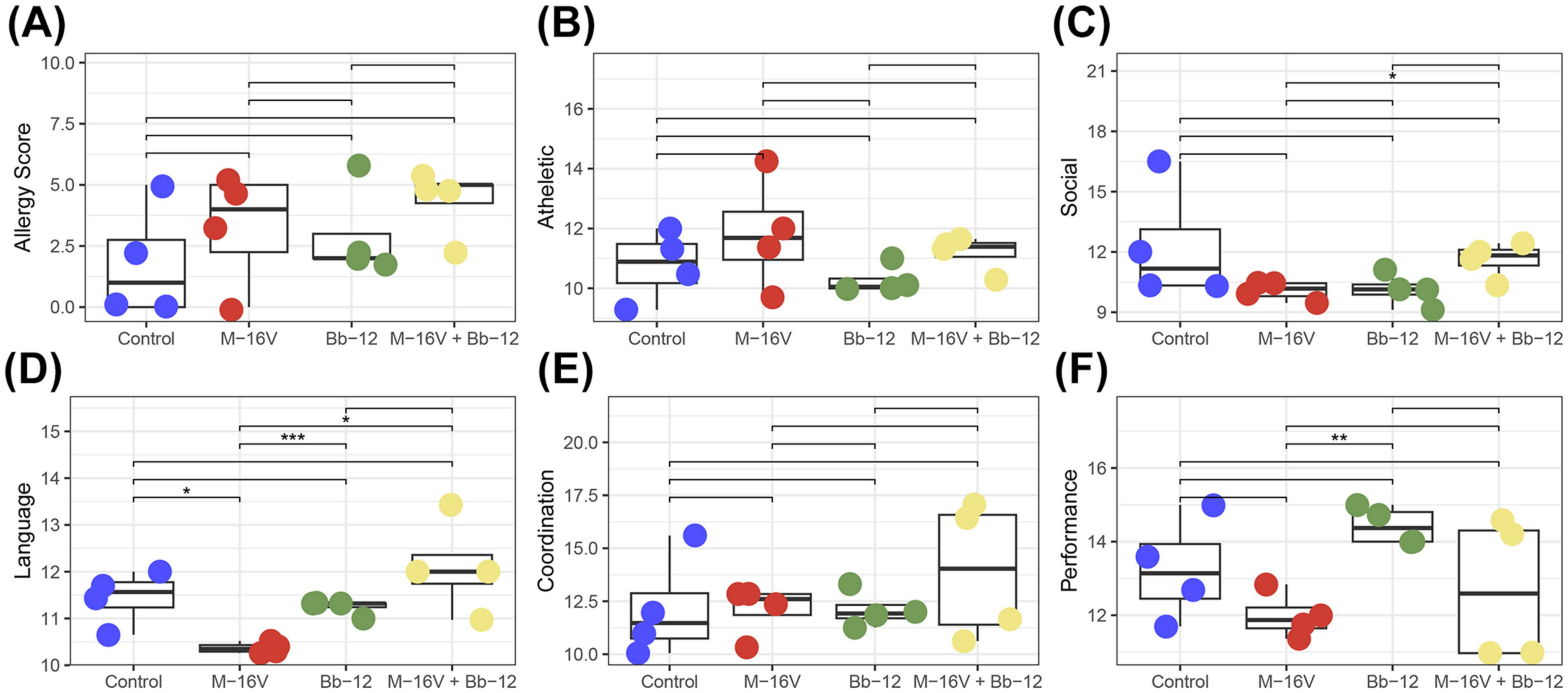
The long-term effects of probiotics on neonates with jaundice after phototherapy were assessed using the Griffith scale. **(A)** Allergy score; **(B)** athletic score; **(C)** social score; **(D)** language skills score; **(E)** coordination score; **(F)** performance score. *p < 0.05; **p < 0.01; ***p < 0.001.

To test the long-term influence of probiotics on neonatal jaundice treated with phototherapy, we followed up with the patients and administered the Griffith test when the children were 1.5–2 years old. The results showed that the Bb-12 group showed better behavior in language and performance, which was significantly higher than the M-16 V group (Figure 3).

### 3.4 Probiotics exhibited considerable anti-inflammatory properties

**Figure 4:**
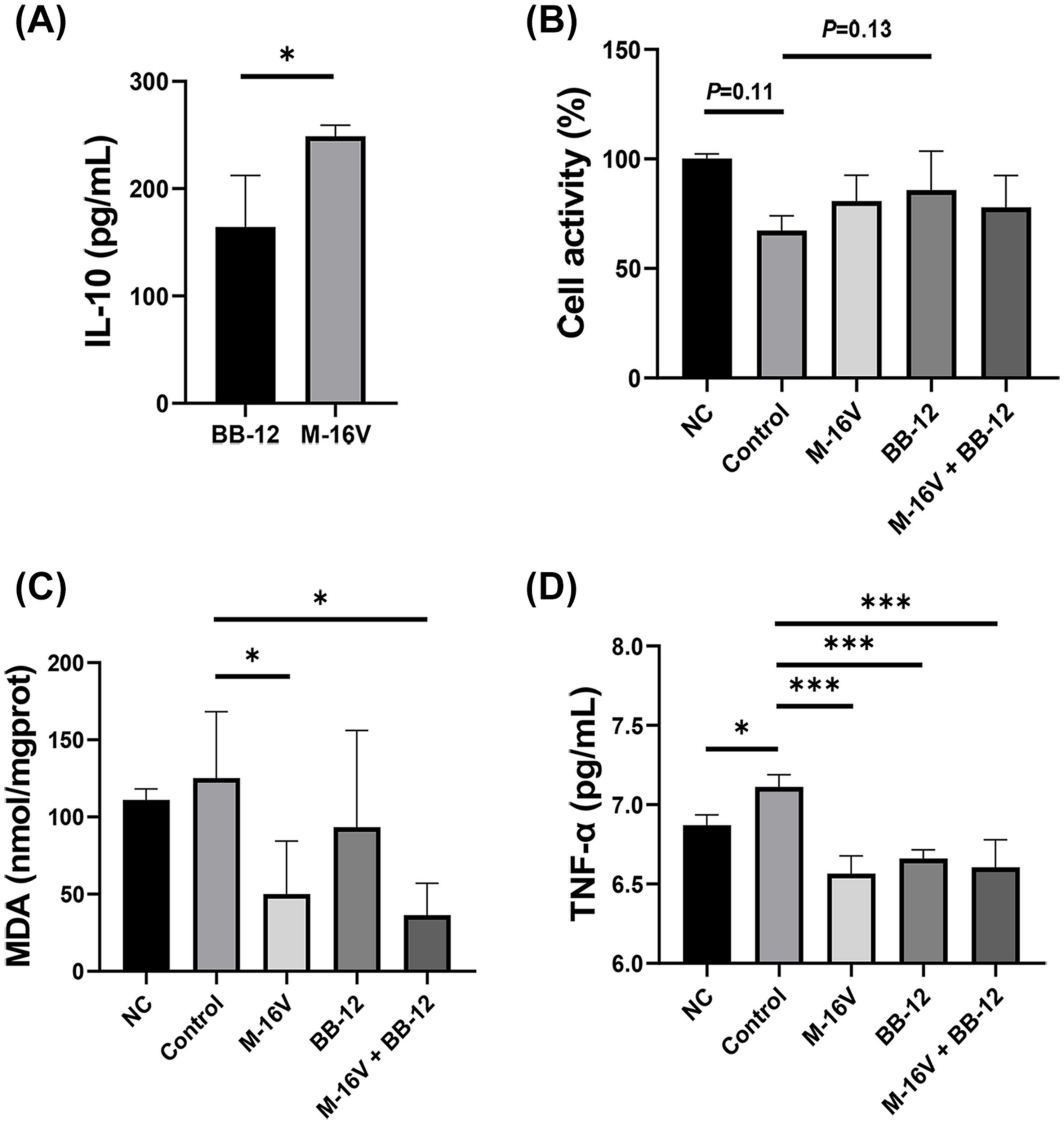
*In vitro* cell culture studies were conducted to investigate the anti-inflammatory potential of M-16 V and Bb-12. **(A)** IL-10 levels; **(B)** Cell activity; **(C)** MDA levels; **(D)** TNF-α levels. The experimental groups were defined as follows: Normal control (NC) group, control (phototherapy group), M-16 V (phototherapy+M-16 V group), Bb-12 (phototherapy+Bb-12 group), M-16 V + Bb-12 (phototherapy + combination group). *p < 0.05; **p < 0.01; ***p < 0.001.

To further validate our findings, we evaluated the anti-inflammatory potential of M-16 V and Bb-12 by measuring IL-10 levels in cell culture. Elevated IL-10 levels were observed following probiotic treatment (Figure 4A). Furthermore, to assess the impact of phototherapy on cell viability, we measured cell activity across different groups. Blue light exposure significantly inhibited cell activity (Figure 4B). MDA levels—which reflect the degree of free radical accumulation and lipid peroxidation—were significantly reduced in the M-16 V and M-16 V + Bb-12 groups (Figure 4C). Besides, levels of pro-inflammatory cytokine TNF-α were significantly increased in the group exposed to blue light alone; probiotic supplementation significantly attenuated this increase (Figure 4D).

### 3.5 Probiotics reshaped the gut microbiota in neonates with jaundice after phototherapy

**Figure 5:**
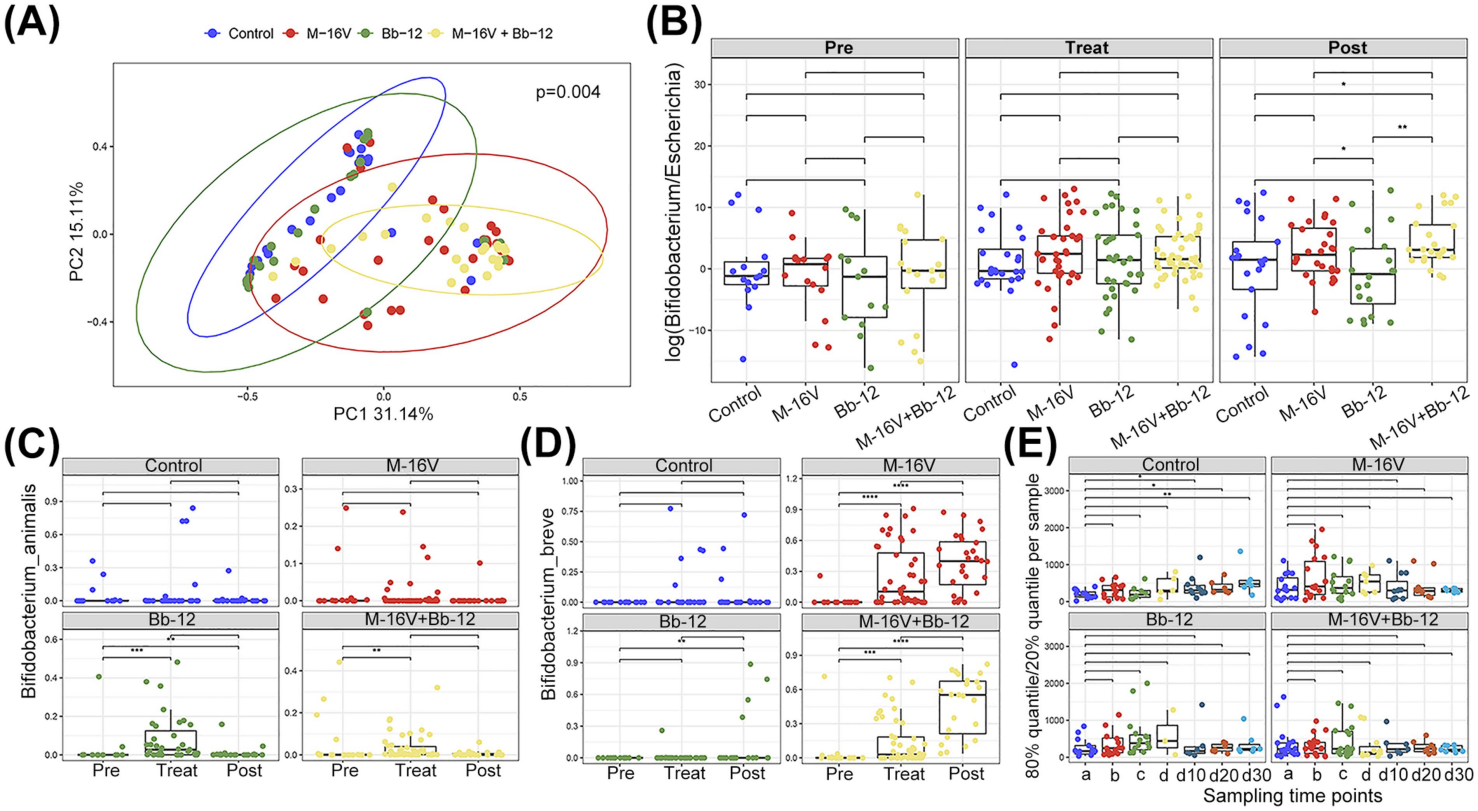
Probiotics altered the beta diversity and the community structure of the gut microbiota. **(A)** Beta diversity of different groups after the phototherapy treatment; **(B)** the ratio of *Bifidobacterium* and *Escherichia* of different groups at different stages; **(C)** *Bifidobacterium animalis* change in different groups; **(D)** *Bifidobacterium breve* change in different groups; **(E)** uniformity of different groups at different stages. *p < 0.05; **p < 0.01, ***p < 0.001.

For beta diversity, we observed significant differences in the gut microbiota structure between the M-16 V group and the M-16 V + Bb-12 group during treatment (Figure 5A). The ratio of *Bifidobacterium* to *Escherichia* is an important index for infant gut health. In our results, compared with the control group and the Bb-12 supplemented group, the M-16 V group and the M-16 V + Bb-12 groups behaved better in this aspect (Figure 5B). It was also observed that the M-16 V supplement does have a better colony outcome than Bb-12 (Figure 5D). The abundance of *B. breve* in the M-16 V group was higher than that in the Bb-12 group. However, the abundance of *B. animalis* in the Bb-12 group was higher than that in the M-16 V group (Figure 5C). Furthermore, we measured the uniformity of gut microbiota (defined as the 80% quantile of 20% quantile of species abundance per sample) across different groups, and found increased uniformity in post-treatment for the control group (Figure 5E).

**Figure 6:**
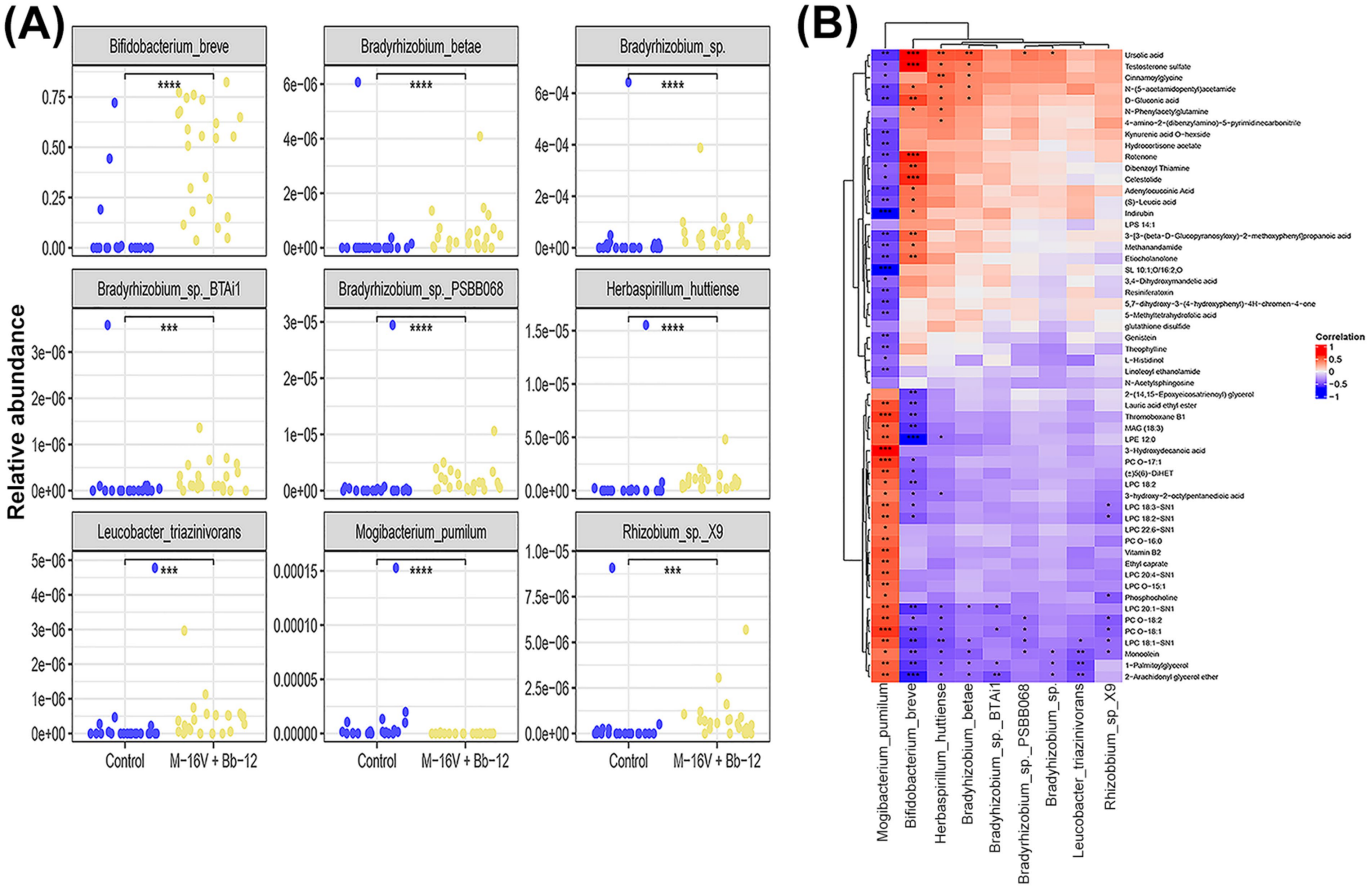
Correlation of differentiated metabolites and species between the M-16 V + Bb-12 group and the control group at the post-treatment stage. **(A)** Differential species between the M-16 V + Bb-12 group and the control group; **(B)** correlation heatmap of differentiated metabolites and species. *p < 0.05; **p < 0.01; ***p < 0.001.

To determine the difference of gut microbiota between groups, especially for the M-16 V + Bb-12 group, we found nine significantly different species that might impact the gut function (Figure 6A). Eight of the species were enriched in the M-16 V + Bb-12 group, while the remaining one, *Mogibacterium pumilum*, was overrepresented in the control group. Finally, we performed a correlation analysis between differential species and metabolites (Figure 6B); we identified *M. pumilum* and *B. breve* as the most correlated species with differential metabolites, suggesting that they may be the driving forces in the evolution of gut function during the post-treatment period.

### 3.6 Probiotic intervention increased metabolites associated with DNA synthesis, growth, and development

**Figure 7:**
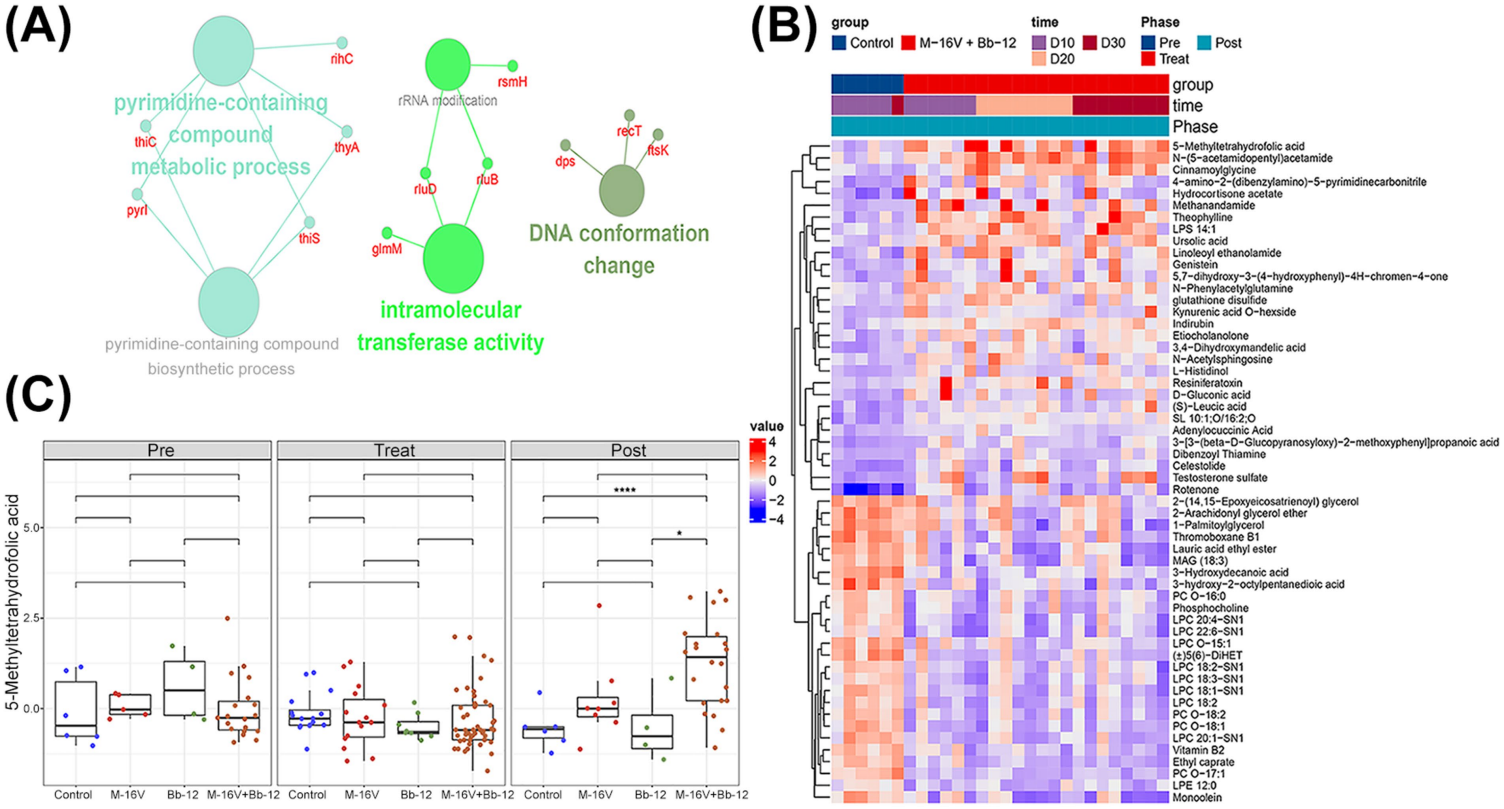
Probiotic intervention changed the metabolites of phototherapy for neonatal jaundice. **(A)** Pathway enrichment analysis of differential genes enriched in M-16 V + Bb-12 group; **(B)** heatmap of differential metabolites between control group and M-16 V + Bb-12 group at visit; **(C)** the abundance of 5-methyltetrahydrofolic acid in different groups at different stages. *p < 0.05; **p < 0.01; ***p < 0.001.

Using related gene for pathway enrichment analysis, we found that pyrimidine-containing compound metabolic process, intramolecular transferase activity, and DNA conformation change were upregulated in the probiotic-supplemented groups (Figure 7A). To further validate this finding, we tested the differential metabolites between the two groups (Figure 7B), among the 55 differential metabolites, 5-methyltetrahydrofolic acid is significantly higher in M-16 V + Bb-12 group at post-treatment (Figure 7C), which has been proven to be the most biologically active form of the B-vitamin (folic acid), and previous studies have reported its crucial role in DNA synthesis.

**Figure 8:**
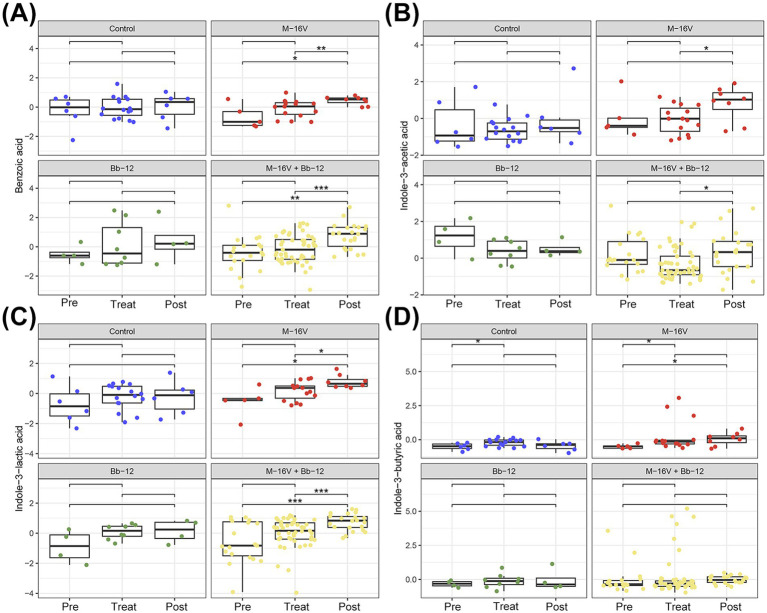
Comparison of benzoic acid, indoleacetic acid, indolelactic acid, and indolebutyric acid before, during, and after treatment **(A–D)**. ^*^p < 0.05, ^**^p < 0.01, ^***^p < 0.001.

We further compared metabolites shown to have an impact on growth and development in previous studies, and found that the M-16 V + Bb-12 group had higher levels of phenylacetic acid (Figure 8A; metabolite related to social behavior), indoleacetic acid (Figure 8B; growth promotion), and indolelactic acid (Figure 8C; inflammation reduction) after treatment compared to before treatment.

## 4 Discussion

In this study, we compared factors potentially affecting the gut microbiota between the probiotic and control groups, including birth pattern, sex, age, maternal age, transcutaneous bilirubin before treatment, gestational age, birth weight, and feeding pattern; no statistically significant differences. Clinically, there are more jaundiced newborns, most of whom need phototherapy, but there is currently no consensus regarding the optimal protocol for probiotic use (Guo et al., 2020; Akagawa et al., 2019; Li et al., 2021).

A meta-analysis of 13 randomized controlled trials evaluating the clinical value of probiotics (including *Bifidobacterium*, *Streptococcus boulardii*, *Clostridium butyricum*, and *Bacillus subtilis*) in the treatment of neonatal jaundice showed a significant improvement in neonatal jaundice not only by reducing total bilirubin, jaundice resolution time but also by shortening phototherapy and hospital stays (Chen et al., 2017). However, these results only mentioned that indicators could be improved, and the extent of the improvement was not specifically analyzed. Results from another systematic review showed that the average duration of phototherapy for neonates with jaundice treated with *Lactobacillius*, bifidobacteria, and yeast was reduced by 11.8 h, TSB was reduced by 1.7 mg/dL, and hospital stay was decreased by 0.32 days (Deshmukh et al., 2019). The data from this study showed that the number of days of hospital stay in the combined probiotic group was reduced by 1.10 days compared with the control group, and the rate of bilirubin decline was increased by 11.3%. A prospective, double-blind, placebo-controlled trial of 119 neonates showed no significant effect of *S. boulardii* on the clinical course of hyperbilirubinemia (Serce et al., 2015). This may be related to probiotic species, survival, and proliferative ability in the intestinal environment, and *S. boulardii* is not a dominant core species in the intestinal tract of infants and young children. The efficacy of probiotics varies across species and even within strains. Much of the evidence regarding the efficacy and safety of probiotic supplementation for neonatal jaundice is of low quality, lacks in-depth studies, and does not clearly indicate a recommendation. In our previous study, we found that the abundance of *Bifidobacterium* in the intestinal flora of jaundiced neonates receiving phototherapy decreased significantly (Fan et al., 2022). *Bifidobacterium* is the most important core species for the development of gastrointestinal microbiota in early infants (Akagawa et al., 2021), which gradually increases with age and becomes the dominant bacterium rather than yeast or lactobacillus. First of all, the difference in the retention rate showed that after the addition of probiotics, they could be quickly colonized in the intestine (Figure 5), the planting effect of M-16 V was better than that of Bb-12, and the abundance of M-16 V in the M-16 V + Bb-12 group was higher than that in the M-16 V group after treatment, which proved that the combined use of probiotics had a synergistic determination effect, and we could further explore the relationship between probiotics and the intestinal flora of jaundiced newborns on the basis of fixed values.

In the past, it was considered that the increased frequency of stool was diarrhea (Faulhaber et al., 2019); it was one of the side effects of phototherapy. This study found that increased stool frequency in the Bb-12 supplemental group may facilitate bilirubin excretion in the stool, thereby reducing jaundice. Increased gastrointestinal peristalsis and accelerated emptying will enhance the children’s appetite and contribute to increased milk intake. Reduce jaundice caused by underfeeding; this may also help increase bilirubin excretion. However, at the same time, clinicians also need to be cautious. Phototherapy-induced intestinal dysbiosis is known to increase stool frequency, which may be further exacerbated by probiotic supplementation. Nursing staff and family members should be informed to provide careful care. The reason for this is that studies have shown that *Bifidobacterium* in the intestinal tract of newborns can convert bilirubin and reduce the reabsorption capacity of bilirubin in the body (Soma and Inagawa, 2015). Our study confirms that M-16 V and Bb-12 supplementation can increase the level of bilirubin in stool, reduce the level of bilirubin in the body, and significantly shorten the duration of phototherapy by approximately 1 day (3.6 days in the experimental group vs. 4.7 days in the control group), and shorten the length of hospital stay for children with jaundice. This reduces certain social and economic costs, and shortens the separation time of mother and child; however, for jaundiced newborns who do not meet the indication of light therapy, more clinical studies are needed to determine the necessity of *Bifidobacterium* supplementation. When M-16 Vor BB-12 were used alone, a decreasing trend in hospital stay was observed, though it was not statistically significant.

Clinical indicators improved after probiotic supplementation; we also need to determine whether the microflora disturbance caused by phototherapy can be reversed by probiotics. Some studies have analyzed the alpha diversity of intestinal microbiota in jaundiced newborns and found that the Chao 1 and Simpson indices were significantly decreased (Duan et al., 2018). Dong et al. (2018) reported that the intestinal flora of neonates delivered by cesarean section was significantly associated with a low risk of jaundice. Our results showed that the combination of M-16 V + Bb-12 could improve the alpha diversity of intestinal microbiota, which had been reduced by blue light treatment in this group. The alpha diversity in the probiotic group was significantly higher than that in the control group. This trend continued until after the third session of phototherapy. Beta diversity analysis revealed distinct clustering patterns between the two groups. Further analysis of the intestinal flora of the M-16 V + Bb-12 group and the control group revealed the main difference to be the increased abundance of specific taxa, including *Bifidobacterium*, *Bradyrhizobium* spp., *Herbaspirillum huttiense*, and *Leucobacter triazinivorans*, as well as an increased abundance of *M. pumilum*. Studies of this bacterium have been associated with oral inflammation (Liu S. et al., 2023). Combined with probiotics may lead to an increased diversity in the microflora, and bacteria leading to an inflammatory response will reduce their diversity; compositional changes in the microbiota are considered a trigger for low-grade systemic inflammation and oxidative changes. These changes may contribute to the development of neurodegenerative and inflammation-related diseases, likely by promoting pro-inflammatory signals and increasing intestinal permeability (the so-called intestinal leakage) (Chen and Yuan, 2020). Probiotics can reduce the disturbance of the microflora caused by jaundice after phototherapy. It may also reduce the inflammatory response caused by the disordered microflora.

Jaundice is an inflammatory response (Lo et al., 2020), which can be aggravated by phototherapy. To investigate whether probiotics can attenuate this inflammation and accelerate jaundice resolution, we conducted additional *in vitro* experiments to assess the inflammatory markers in each group. This study suggests that the probiotic addition group exhibited lower levels of inflammatory factors (MDA and TNF-α). These findings indicate that probiotics can alleviate microflora disorders and the consequent inflammatory response caused by phototherapy. It has been shown that probiotics also contribute to goblet cell barrier integrity by increasing mucin expression and secretion, thereby limiting bacterial movement in the mucus layer. They can also promote the secretion of secretory IgA (sIgA) into the luminal mucus layer, thereby supporting intestinal homeostasis (Ohland and Macnaughton, 2010). Moreover, some probiotics can compete with pathogens or symbionts for binding sites on mucins or epithelial cells by expressing antimicrobial factors, such as bacteriocins, thereby preventing colonization by harmful bacteria and promoting barrier function, and directly killing or inhibiting the growth of pathogenic bacteria (Rahman et al., 2024). This is consistent with the results of this study that the probiotic addition group reduced the abundance of *Escherichia coli*. Immune cells and bacterial components [such as lipopolysaccharide (LPS)] can escape the inflamed intestine and enter the circulation, and the resulting systemic low-grade inflammation can eventually drive neuroinflammatory responses through various routes. The study by Yang et al. (2019) also shows that probiotics can inhibit LPS-induced NF-κB activation at the bacterial surface, reduce the expression of inflammatory factors, such as TNF-α, IL-8, cell adhesion molecule 1, and cyclooxygenase-2, reduce cell apoptosis, and inhibit the body’s inflammatory response. Systemic inflammation can impair BBB integrity, allowing foreign molecules to enter the brain, triggering the release of cytokines, activating the proinflammatory potential of microglia and astrocytes, and initiating neuroinflammation and disruption of neurons and neural processes. This is consistent with the results of our study, finding that *Bifidobacterium* was able to reduce TNF-α; this further demonstrates that probiotics play a positive role as a regulator of microbiome indicators, enhance the stability and diversity of intestinal flora, supplement probiotics reduced by phototherapy, reduce the abundance of conditional pathogenic bacteria, and improve the effect of inflammation. Our Griffith assessment at 1.5–2 years found that the Bb-12 group performed better in language and performance, confirming that bifidobacteria mitigated the impact of inflammation on the nervous system. However, due to family refusal to follow up, in this study, only about five children could be recalled in each group during the Griffith evaluation. This may have contributed to the failure of the combined probiotic group in nervous system development.

We previously found that MDA levels, a marker of free radical accumulation and lipid peroxidative damage, were significantly lower in the M-16 V and M-16 V + Bb-12 groups than in the control group. In addition to repairing inflammatory damage, we further found that the levels of metabolites involved in DNA damage, such as 5-methyltetrahydrofolate (cell repair), phenylacetic acid (a metabolite related to social behavior and anti-inflammatory substances), indoleacetic acid (promoting growth), and indoleacetic acid (decreased inflammation), were significantly higher in the M-16 V + Bb-12 group than before phototherapy. Folate is one of the important nutrients supporting physiological functions and DNA synthesis in the nucleus. Low folate levels are a risk factor for several diseases, including cardiovascular disease and neural tube defects. Folate metabolism to bioactive tetrahydrofolate requires several enzymes and cofactors, while 5-methyltetrahydrofolate is used directly and is involved in one-carbon metabolism, and the use of 5-methyltetrahydrofolate as an alternative folic acid supplement is currently increasing worldwide (Obeid et al., 2013). Bifidobacteria have been studied as a source of host folate, and unabsorbed dietary vitamins (such as folic acid) can serve as growth factors for colonic bacteria. They may help to promote the growth of beneficial intestinal bacteria while inhibiting harmful bacteria, regulate the host’s immune cell function, and improve the ability of damage repair (He and Li, 2023; Houghton et al., 2006; Scaglione and Panzavolta, 2014). The 5-methyl folate group was more abundant, suggesting that *Bifidobacterium* may reduce DNA damage caused by phototherapy by promoting folic acid synthesis. Phenylacetic acid is one of the most common natural auxin growth regulators in plants, which has been shown to have simultaneous anti-inflammatory effects (Kwon et al., 2024). As derivatives of both acetate and lactate, they also have growth-promoting and anti-inflammatory effects (Tomberlin et al., 2017). Studies have shown that indole induces the expression of the anti-inflammatory cytokine receptors (IL-10 and IL-11). Furthermore, exposure to indole decreased expression of several other pro-inflammatory cytokines in HCT-8 cells, thereby inducing coordinated changes in expression of pro-inflammatory and anti-inflammatory cytokines and chemokines. It also reduced TNF-α-induced IL-8 production by 1.8-fold over 18 h, clearly demonstrating indole’s ability to mitigate TNF-α-mediated inflammation in HCT-8 cells (Bansal et al., 2009). These findings demonstrate that it can serve as a beneficial interkingdom signal that improves intestinal epithelial cell function, control inflammation, and increases resistance to pathogen colonization. This is consistent with the results of this study, in which the anti-inflammatory effects of indole and its derivatives accounted for the lower levels of the pro-inflammatory factor TNF-α and the higher levels of the anti-inflammatory factor IL-10 in the probiotic supplementation group. This further supports the hypothesis that the anti-inflammatory effect of probiotic addition may be mediated by metabolites.

However, several limitations must be acknowledged. First, the sample size was relatively small, and the study was conducted at a single center, which may limit the generalizability of the results. Second, we lacked a parallel healthy control group for microbiome comparison. Third, stratified randomization based on feeding mode was not performed at enrollment. This is notable given that feeding patterns significantly influence both neonatal gut microbiota establishment and the clinical course of jaundice (Bratton et al., 2023; Inchingolo et al., 2024). To mitigate this potential confounding effect, we applied a Generalized Linear Model (GLM) to adjust for feeding mode. The adjusted analyses revealed that M-16 V + Bb-12 continued to significantly promote *B. breve* colonization and reduce hospital stay duration, independent of feeding patterns (see Table 1). Nevertheless, future multi-center clinical trials with larger cohorts and healthy control groups are warranted. These studies should incorporate stratified analyses of covariates—such as feeding mode, delivery mode, and gestational age—to further validate these therapeutic effects.

## 5 Conclusion

Our study demonstrates that the supplementation with *Bifidobacterium* strains (M-16 V and Bb-12) is a safe and effective therapeutic strategy for newborns with jaundice undergoing phototherapy. This probiotic combination effectively modulates the intestinal environment by enhancing microbiota diversity and reducing the abundance of potentially harmful bacteria. Mechanistically, it facilitates bilirubin excretion by increasing stool frequency, thereby lowering serum bilirubin levels and alleviating clinical symptoms. Additionally, the intervention helps repair mucosal damage and relieve inflammation caused by phototherapy and jaundice, thereby offering potential protection against inflammation-induced neurological injury.

## Data Availability

The data presented in this study are publicly available. The data can be found in the EBI MetaboLights repository (accession MTBLS14123) and the NCBI Sequence Read Archive (SRA) (accession PRJNA1425751).

## Footnotes

1
https://www.mzcloud.org/

2
https://www.genome.jp/kegg/pathway.html
